# Understanding effective care management implementation in primary care: a macrocognition perspective analysis

**DOI:** 10.1186/s13012-015-0316-z

**Published:** 2015-08-21

**Authors:** Jodi Summers Holtrop, Georges Potworowski, Laurie Fitzpatrick, Amy Kowalk, Lee A. Green

**Affiliations:** Department of Family Medicine, University of Colorado Denver School of Medicine, 12631 E 17th Avenue, Mail Stop F496, Room 3505 Academic Office 1, Aurora, CO 80045 USA; Department of Health Policy, Management, and Behavior, School of Public Health, University at Albany, State University of New York, Albany, NY USA; Department of Family Medicine, Michigan State University College of Human Medicine, Grand Rapids, MI USA; Priority Health, Grand Rapids, MI USA; Department of Family Medicine, University of Alberta, Edmonton, Alberta Canada

## Abstract

**Background:**

Care management in primary care can be effective in helping patients with chronic disease improve their health status. Primary care practices, however, are often challenged with its implementation. Incorporating care management involves more than a simple physical process redesign to existing clinical care routines. It involves changes to who is working with patients, and consequently such things as who is making decisions, who is sharing patient information, and how. Studying the range of such changes in “knowledge work” during implementation requires a perspective and tools designed to do so. We used the macrocognition perspective, which is designed to understand how individuals think in dynamic, messy real-world environments such as care management implementation. To do so, we used cognitive task analysis to understand implementation in terms of such thinking as decision making, knowledge, and communication.

**Methods:**

Data collection involved semi-structured interviews and observations at baseline and at approximately 9 months into implementation at five practices in one physician-owned administratively connected group of practices in the state of Michigan, USA. Practices were intervention participants in a larger trial of chronic care model implementation. Data were transcribed, qualitatively coded and analyzed, initially using an editing approach and then a template approach with macrocognition as a guiding framework.

**Results:**

Seventy-four interviews and five observations were completed. There were differences in implementation success across the practices, and these differences in implementation success were well explained by macrocognition. Practices that used more macrocognition functions and used them more often were also more successful in care management implementation.

**Conclusions:**

Although care management can introduce many new changes into the delivery of primary care clinical practice, implementing it successfully as a new complex intervention is possible. Macrocognition is a useful perspective for illuminating the elements that facilitate new complex interventions with a view to addressing them during implementation planning.

## Introduction

Chronic care management is a team-based, patient-centered approach to addressing the complex health care needs of individuals with chronic illness. This strategy aims to engage patients in “activities designed to assist patients and their support systems in managing medical conditions more effectively” [1]. Care management often involves adding the role of care manager, usually a nurse or social worker by training, to the practice team. Care managers perform functions such as educating patients about their chronic conditions, motivating patients to improve their health behaviors, referring patients to resources for additional support, and coordinating care over time and across care settings. Research on care management demonstrates that it can be effective in helping patients improve their clinical health indicators (e.g., blood pressure, hemoglobin A1c) and reduce complications of their disease [2–4]. In the USA, it is a central element of the transformation of primary care to the Patient-Centered Medical Home model [5–8].

Work by our team as well as other investigators shows that care management can be highly variable in terms of what is conducted, who conducts it, and how well it is implemented; thus it varies in effectiveness [9, 10]. Implementing care management can be a challenge because it can require new staff, new physical and cognitive workflows, new assessment tools, and new connections to resources [6, 9, 11]. Embedding care managers to work on site at the practice in collaboration with practice staff, allowing for more integrated care, appears to be a characteristic of effective care management [9, 12, 13] but practices vary in how, and how well, they accomplish it.

Research to date on implementation of care management has tended to describe broad characteristics of settings that were successful or not successful, such as the size of the practice or patient characteristics, or report on broad-brush barriers such as lack of time and money. Our research team sought to gain a richer understanding of implementation, at a more detailed level, to help inform an actionable strategy of what it takes to effectively implement care management.

Our approach was to apply the macrocognition framework [14, 15]. Macrocognition is defined as the study of thinking as it occurs in the performance of complex, real-world tasks. The concept and study of macrocognition is contrasted to “microcognition,” which is the more controlled study of isolated elements of cognition in laboratory conditions, such as the study of working memory using contrived tasks [16, 17]. The study of macrocognition arose out of the realization that research on microcognition was of very limited usefulness to real-world decision makers and teams [17]. The macrocognition framework focuses on the cognitive components of, and skills needed to accomplish actual knowledge work (Table 1). It also explicitly recognizes that the knowledge of individual experts and expert teams is often neither observable nor readily accessible to introspection.

**Table 1:** Macrocognition-coding glossary

Macrocognition function and “Code” assigned	Definition
Sensemaking and searning (SL)	A deliberate and systematic attempt to find coherent, conceptual *situational* understanding, acquire new knowledge, or generate shared mental models
Decision making (DM)	Any decision *in* the clinical process, including what decision, by whom, made how, when, where, and why about an individual patient’s care management
Planning (PL)	Any activity involving the process of intending to (re-)shape another process, e.g., decisions *about* the clinical process. Planning (about) something, including learning, coordinating, etc. Includes re-planning
Monitoring and detection (MD)	Tracking implementation progress or discovering a situation that is novel, or a potential opportunity or problem, or deviations from expected processes or outcomes
Managing the unknown, uncertain, unexpected, and irregular (MU)	How uncertainty, risk, and ambiguity are dealt with, including identification of ambiguities and risks, monitoring strategy, and incorporation into decision making; dealing with inadequate information
Coordinating (CO)	Any activity that helps synchronizes two or more people involved in an activity, about clinical and change process; developing and maintaining common ground (often in planning or sensemaking)

The primary toolset for applying the macrocognition framework is cognitive task analysis (CTA) [16, 17]. CTA is a set of highly structured and complementary qualitative or quantitative methods drawn from diverse fields of study, such as anthropology and ergonomics. Each type of CTA is designed to elicit the details of one or (more typically) several of the macrocognitive functions listed in Table 1, in real-world environments. The choice of CTA method depends on context. For example, the Critical Decision Method would be applied to investigate a team’s coordination, sensemaking, etc. in an unusual occurrence such as a near-miss event in surgery, while the Team Knowledge Audit would be chosen to develop a deep understanding of how these macrocognition functions play out in a surgical team’s routine operations. The macrocognition framework with its CTA toolset offers the advantage of a decades-long track record of successfully understanding and guiding the improvement of individual and team performance in a range of complex knowledge work settings where failures would be both very visible and costly, such as aviation, nuclear power plant operations, and thoracic surgery [17–22]. Our team pioneered the use of CTA in primary care [23], using it to understand how physicians structure visits. In this project, we applied CTA to understanding in detail how practices implemented chronic care management, and why implementation went well in some, but poorly in others.

In this paper, we apply the macrocognition framework to the understanding of care management implementation in primary care. Our questions include the following: (1) Does the macrocognition framework, and the use of its associated tools, provide a way to illuminate processes that practice teams engage in related to implementing care management, and (2) is how, and how well, practices carry out these macrocognition processes associated with the reach and effectiveness of their care management efforts?

## Methods

### Study context

This explanatory study of implementation using cognitive task analysis (CTA) was one component of a mixed-methods randomized controlled trial of implementing a specific approach to chronic care management, the chronic care model (CCM), for diabetes management and prevention in a set of primary care practices. The CCM is a framework for chronic disease care that includes self-management support, delivery system design, decision support, and clinical information systems [24, 25]. The implementation of the CCM included the key element of care managers embedded in practices. Prior to this initiative, participating practices did not have a care manager. Beyond the CCM framework, practices were given the flexibility to implement care management in a way that fitted best for their practice. Care managers (1) developed a practice plan for CCM implementation in collaboration with the clinical team, (2) provided chronic disease self-management and health behavior change assistance through patient counseling and referrals, (3) coordinated care with health care and other resources, and (4) tracked, collected, and reported patient data.

Table 2 provides an overview of all of the methods and analysis steps conducted for the study. Institutional review board approval for the study was received at Michigan State University and the University of Michigan.

**Table 2:** Methods sequence

1. Sampling and preparation
i. 10 practices selected
ii. Practices paired by specialty and size
iii. One practice from each pair randomly assigned to CMgt condition
iv. Interview guide constructed
2. Data collection: baseline (pre-intervention)
i. Semi-structured interviews with care managers and practice members
ii. Observation (30–120 min) during visit
iii. Practice summary report generated after visit
iv. Summary report member checked
v. Interviews transcribed, cleaned and formatted in Atlas.ti
3. Data collection: interim
i. Each care manager interviewed three times between baseline and follow-up
4. Data collection: follow-up
i. Same process as Baseline data collection (9 months post intervention start)
ii. Outcomes data collected for RE-AIM (16 months post intervention start)
5. Analysis: macrocognition
i. Development of macrocognition-coding guide (a priori categories)
ii. Initial coding by team members, coding calibration, and then coding completion
iii. Quotation outputs generated by practice by code
iv. Independent evidence table constructed over several team meetings
v. Team met to reconcile all evidence tables and themes
vi. Team members independently rated practices on how well and often they engaged in each macrocognition process
vii. Team members independently assigned each practice an overall implementation score
viii. Team met to reconcile macrocognition and implementation scores
6. Analysis: RE-AIM
i. Data (quantitative) for reach, effectiveness, adoption and maintenance of RE-AIM analyzed by practice
ii. Data for implementation part of RE-AIM created by independent ratings and reconciled by qualitative team members
7. Analysis: Outcomes
i. Overall themes related to use of macrocognition processes
ii. Care management implementation success (RE-AIM) by practice
iii. Use of macrocognition processes by practice
iv. Comparing implementation success with use of macrocognition processes by practice

### Design and participants

Ten practices from one physician-owned group practice organization participated in the overall study. Practices were arranged into pairs by specialty (family or general internal medicine) and size (large or small). One practice from each pair was randomly assigned to balance selected practices on these characteristics. Table 3 describes the characteristics of the ten practices. Data for this qualitative report was collected from the five intervention practices.

**Table 3:** Practice characteristics by matched pair

Practice	Specialty	Location	Size^a^
A	Internal medicine	Urban	Small
G 1	Internal medicine	Urban	Small
B	Family medicine	Suburban	Large
F 2	Internal medicine	Suburban	Large
C	Internal medicine	Suburban	Large
I 3	Family medicine	Urban	Large
D	Family medicine	Rural	Small
J 4	Family medicine	Rural	Small
E	Family medicine	Suburban	Medium
H 5	Family medicine	Urban	Medium

Practices designated by letters are intervention practices, and numbers

^a^ *Small* three or less providers, *Medium* four to six providers, *Large* seven or more providers

### Data collection

A semi-structured interview guide for the cognitive task analysis (CTA) “team knowledge audit” method” [16] was constructed, to gather data about the macrocognitive processes involved in each practice’s (a) clinical management of diabetes and pre-diabetes and (b) approach to the implementation of care management. The present analysis focuses on the implementation process.

The interview probes covered a number of topics important to developing a description of the care management program, including who was involved (personnel assignments, and care manager background, training, and role comfort), how care management was introduced, what training and support was provided, what tools and resources were utilized, and thoughts and perceptions about the program. We began by asking each interviewee to describe broadly how care management was introduced to and implemented in the practice, and then probed for several aspects of the implementation process. For example, we asked about who made the key planning decisions about implementing care management and how, and who else was involved and how. We also probed about how practice members learned about and came to understand care management and how it would be implemented, how changes were communicated at each step of implementation process, whether and how feedback was solicited, and on what topics (e.g., ideas for workflow tweaks, changing roles, what was working and what was not). We probed about anticipated implementation failures or problems, whether and how the practice planned to avoid or prepare for them, detect them when they arose, and learn from them. We also probed about how implementation success was assessed and how the practice adapted when existing processes were not satisfactory.

We used the guide to interview practice members during site visits at two time points: baseline (just prior to the intervention beginning) and 9 months later to allow practices sufficient time to implement and to have begun routinizing care management. Generally, the same individuals were interviewed at both time points, although some differences in follow-up interviews occurred due to scheduling. Two researchers (a co-investigator and a research assistant (RA)) visited each practice and conducted each interview together. One researcher led the interview while the other took notes and occasionally asked clarification and follow-up questions. At each practice, interviews were conducted with the five to seven individuals who played key roles in care management. These typically included the care manager, one or two physicians, a medical assistant, the practice manager, and often a clinical supervisor, nurse, or reception staff member. Individuals were selected for interviews by practice leadership and were felt to be sufficient in number to represent the care management program. In addition, the five care managers were interviewed at three more time points between the baseline and early intervention. Each interview lasted from 40 min to 2 h, and a total of 74 interviews were conducted.

During each site visit, RAs also conducted observations of the practice that lasted from 30 min (small practices) to 2 h (larger practices). Field notes were collected using a structured observation template to describe the physical environment, practice personnel and culture, and patient population. After each visit, RAs completed a one-page summary report, which described key findings. We then conducted member-checking by providing each practice with this summary report to receive corrections, which were minimal. Revisions were made based on feedback received. Interview data were audio-recorded and transcribed verbatim. Transcripts were cleaned, formatted, and placed into the ATLAS.ti qualitative software program (version 6; Scientific Software Development, GmbH, Berlin, Germany).

Outcome data were collected using the RE-AIM framework, a well-established program evaluation framework for measuring the different dimensions of implementation success [26, 27]. The multiple criteria for implementation success embodied in the RE-AIM framework provide a rich and nuanced understanding of care management implementation success. At the 16-month mark, reach was measured by the number of patients who were referred to the program, which was adjusted by the full-time equivalency of the care management effort per practice; effectiveness was measured by the improvement the patients made to their health behaviors and clinical values; adoption was measured by participation in referrals by practice providers; and maintenance was measured by patients’ participation in follow-up calls. Implementation was derived qualitatively by the research team as explained below.

### Analysis

Because we were interested in how practices’ macrocognition processes related to their implementation success, we specifically sought out this information in our qualitative analysis. Five members of the research team met regularly to develop a macrocognition-coding guide that contained clearly stated definitions of the macrocognition processes presented in Table 1 and illustrative examples for each, including examples that warranted double-coding. Interviews were then coded using this guide. Three of the five team members coded the same interview transcript then reconciled how they had coded. This was repeated twice more with new transcripts. Each time, inter-rater reliability was evaluated using a reconciliation table. When the three coders reached near-complete agreement on the reconciliation table, they were each assigned their own interview transcripts and completed the coding of the remaining transcripts.

Quotation outputs were generated for each of the six macrocognition codes and then organized by practice. An evidence table spreadsheet was created with four columns: time (whether the quote was from a baseline or follow-up interview), macrocognition code, a narrative description summarizing that macrocognitive process in that practice at that time, and evidence (the line numbers of supporting or disconfirming quotes in the quotation output). All team members read through all of the quotations for the first practice independently. The researchers met three times to present evidence and develop the narrative description for each code for that practice. Next, the analysis team members divided up the remaining practices and worked in teams of two to complete the evidence tables for those practices. The evidence table analysis meetings lasted 90 min each and were held two or three times monthly for 6 months.

After evidence tables had been completed for all practices, the research team members used them to independently rate each practice on how well (and how often) it used each of the macrocognition processes on a 4-point ordinal scale: 4 = used well and often; 3 = used well, but not often; 2 = a mix of used well and not well; 1 = not used or not used well. The team then met to reconcile raters’ scores, which were usually the same or off by one point. Discrepancies in scores were resolved through discussion, and consensus was reached for each final score. These ratings become part of the cells that are later described in Table 6 (and correspond to the symbols in that table).

To address our second research question, the team needed to complete all the components of the RE-AIM framework for the composite measure of care management program success. Table 4 outlines the elements of RE-AIM and how they were attributed to our study. As noted above in the final paragraph of the data collection, most the RE-AIM components (except “I” for implementation) were quantitatively collected. These results were analyzed by a separate part of the research team responsible for quantitative analysis. To assess implementation, the qualitative research team members rated each practice independently on their overall implementation success by assigning a rating of excellent, good, fair or poor, and then met to reconcile those ratings.

## Results

### Care management implementation success

In the parent study, the quantitative analysis demonstrated that care management intervention patients, compared to matched patients in comparison practices, improved on the two main outcomes targeted for the intervention: better hemoglobin A_1_c control for diabetic patients (adjusted mean difference in difference 0.16 for A1c < 8; CI 0.08, 0.23) and weight loss in non-diabetic patients (adjusted mean difference in difference 0.18 for weight loss >5 %; CI 0.08, 0.29).

In the present explanatory study, we examined the five practices for implementation success based on the RE-AIM definitions that were outlined in Table 4. Table 5 provides the results for each practice’s outcomes for each of the RE-AIM elements. For ease of understanding, the practices are labeled from A through E with A being the most successful with implementation, B the second most successful and so on, though practice E as the least successful (this was not their order of implementation or interview visits). All practices were similarly effective at clinical changes, or effectiveness. However, there were clear differences by practice in the number of patients per full-time equivalent care manager in terms of participating (reach), implementation, and follow-up assessment completion (maintenance). Adoption of care management as assessed by the number of accepted referrals did not vary widely among practices. There appeared to be differentiation in overall implementation success, with practices A and B scoring higher than the other practices on nearly all RE-AIM assessment points. Practice C was in the middle, and practices D and E were lowest ranking in almost all areas, especially practice E.

**Table 4:** Determining practice implementation success using RE-AIM

RE-AIM element	Description of element^a^	How assessed in this study (per practice)
Reach	The absolute number, proportion, and representativeness of individuals participating in an initiative	Number of patients enrolled per FTE care manager
Effectiveness	The impact of an intervention on important outcomes, including potential negative effects, quality of life, and economic outcomes	Improvement in clinical values for patients in CM
Adoption	The absolute number, proportion, and representativeness of settings and intervention agents who are willing to initiate a program	Distribution of providers referring to CM
Implementation	At the setting level, implementation refers to the intervention agents’ fidelity to the various elements of an intervention’s protocol. This includes consistency of delivery as intended and the time and cost of the intervention	Rating given from review of interview data regarding (1) knowing how to use the program, (2) reported use, (3) meaning and value, and (4) enthusiasm and support
Maintenance	The extent to which a program or policy becomes institutionalized or part of the routine organizational practices and policies. Maintenance in the RE-AIM framework also has referents at the individual level. At the individual level, maintenance has been defined as the long-term effects of a program on outcomes after 6 or more months after the most recent intervention contact	Patient follow-up completion rates

^a^From www.RE-AIM.org

**Table 5:** Practice RE-AIM success outcomes

Practice	Reach^a^	Effectiveness^b^	Adoption^c^	Implementation^d^	Maintenance^e^	Overall outcome rank-order
A	290 FTE	Good	3/3	Good	70.3 %	1
B	241 FTE	Good	6/6	Good	52.1 %	2
C	189 FTE	Good	7/8	Fair	40 %	3
D	125 FTE	Good	2/4	Fair	48 %	4
E	94 FTE	Good	6/8	Poor	38 %	5

^a^Reach refers to the number of patients who received care management per FTE care manager

^b^Effectiveness refers to the behavior change and clinical improvements made by patients participating in care management

^c^Adoption refers to the proportion of providers referring 5 or more patients to the care manager

^d^Implementation refers to a qualitatively derived rating for the implementation of care management

^e^Maintenance refers to the 6-month follow-up rate of patients with the care manager for that scheduled assessment

### Use of macrocognition functions

Keeping the same ranking and order from A to E from the care management implementation success determined above, we included the rankings for each of the macrocognitive functions (noted in Table 1) on our ordinal scale in Table 6. The overall pattern was that practices that had effective processes in place for planning, coordinating, decision making, sensemaking and learning, problem monitoring and detecting, and managing the unknown were the same practices that were successful at care management implementation across the RE-AIM elements. This was the case for practices A, B, D, and E. Practice C did not follow the pattern as consistently and ranked lower on the macrocognitive processes than did practice D.

**Table 6:** Use of macrocognitive functions and process by practices

Practice	Coordinating	Planning	Decision making	Monitoring and detecting	Managing the unknown	Sense making learning
A	++	++	+	++	++	+
B	++	++	++	++	+	++
C	±	+	±	+	+	±
D	++	+	**+**	+	+	++
E	−	−	±	−	±	−

++ used well and often, + used well, but not often, ± used well and not well, − not used or not used well

### Coordinating

Coordinating is any activity that helps synchronize two or more people involved in an activity about clinical and change process. According to Klein [28] coordination is “the way the team members orchestrate the sequencing of their actions to perform a task.” For the purposes of this study, coordinating focused on clinical or change-related tasks. There are several areas in care management implementation where coordination is needed. Some of these include identification of patients who might be eligible for care management and then communicating to those who are to offer the care management, or if a new health problem or issue is identified by the care manager that the physician needs to know about to attend to with the patient.

The key features of effective coordination that emerged were the amount and quality of the communication among practice team members, and the sense of sharing the care of the patient. Where care managers had very open communication with providers and staff, it was described as flexible and occurring through multiple channels, such as planned huddles, e-mail, and impromptu conversations. This was first evident in how practice members worked together to define the new workflows associated with care management:

*“I came up with some workflows that would try to help get the staff to learn about care management and how to refer to the care manager. I worked on getting those kind of perfected within the leadership team, and then presented those to the providers at the provider meeting and the staff at the staff meetings … Then it was kind of that process that those had to be tweaked throughout, so…going back for feedback individually from providers and staff at meetings, asking for feedback on how things are going, trying to take some of the things that were barriers or difficulties to doing it, and…talking about it again within the leadership team and readdressing it at meetings again to try to come up with different workflows.”* [Care manager from practice B]

Evidence of coordinating was realized in the way the patient care was shared and how each person on the team played a role in doing their part in the care process. Facilitators of effective coordination included the ability of the care manager, providers, and staff to work physically near one another, see patients together in a joint visit, have effective and multiple channels of communication, share common tools that build off one another (e.g., disease template in the electronic medical record; EMR), and having structures for discussion (e.g., patient case conferences or huddles).

Conversely, when care management coordination was lacking, it manifested as a “siloed” operation of patient care. The care manager in those practices operated more independently of the primary care provider to identify patients eligible for care management, call patients to invite their participation, work with patients separately, and then document her notes separately in the EMR. Physical barriers to interaction, such as a care manager sitting away from providers, appeared to make coordination more difficult. When care managers did not offer multiple methods of communication according to provider preference, this also seemed to impair coordination. Providers and other staff members not reading the care manager notes regarding patient care in the EMR was both a type of poor coordination and hampered subsequent coordination.

### Planning

Planning is any activity involving the process of intending to shape or re-shape another process. This includes actions and decisions about shaping the clinical process itself (as opposed to clinical decisions within it). It often incorporates other macrocognitive processes, such as coordinating, sensemaking, or monitoring and detection.

Planning was most evident at the start of the implementation, where the practice teams needed to make decisions about which patients they would deem eligible, how they would offer care management to the patients, where and when patient meetings with care managers would occur, and how communication and documentation would flow between the provider and care manager. As the implementation progressed, the more successful practices engaged in re-planning after considering what was working and what needed improvement and additional planning was needed for new workflows.

Practices that did well in planning had a deliberate approach where lots of ideas were generated, input welcomed, and feedback provided between the leadership team and other staff. Different disciplines were represented to provide diverse points of view, but not such a large group that progress was stalled.

*“We did have an all-staff meeting. Everybody that was involved in the Lean project they went over the board with us, because we did have some clerical coach as well, just to go over the steps as far as the check-in process, getting the patient here for an appointment, getting them checked in; The MA’s role, the doctor’s role, the checkout role; if they needed referral somewhere, so we had an all staff meeting about that and we all came back to look at the board to see each process, and what was changed, and what we could do to better the patient’s visit.”* [Medical assistant from practice C]

Effective practices also incorporated well-considered goals and outcomes for what success would look like. They set and kept regular meetings for key decision makers within the practice to work together. They made the time for conversation that allowed individuals to determine steps for how the care management was going to work, who was involved, and how to know if it was working.

Team members who were effective planners had a solid sense of how the clinical flow could be modified to accommodate care management, and were willing to consider alternatives to make it work (e.g., adjusting who got what roles). This was particularly true for the care manager. Within the practice team, individuals with a sense of systems thinking and knowledge about how clinical processes could function were also helpful.

Staff turnover undermined effective planning and re-planning. The practice manager was often a key member of planning new interventions, and in several practices, the practice manager left during the implementation phase. This led to planning meeting cancelation, meetings without structure or clear agendas, poor meeting facilitation, loss of process expertise, and loss of network connections to others within the larger organization who could serve as advisors for the planning process.

Lack of clear leadership (either from a physician or the practice manager) also led to low planning function. Where this occurred, the care managers were put into the roles of needing to figure out a plan on their own and trying to communicate it to individual practice members or have ad hoc (i.e., hallway) conversations to piece together a plan of implementation.

“*I don’t really ever use that word planning in this office. Things seem very willy-nilly. One of the things is meetings get canceled, or meetings get canceled and I don’t know they’re canceled. We don’t have a meeting room. For example, N and I have tried to meet with our practice manager. We ended up meeting with her once, but it was not our meeting. The one and only meeting that I’ve had with this practice manager we met in her office. We’re like all sitting right on top of each other.”* [Care manager from practice C]

### Decision making

Decision making includes any decision about an individual patient’s care management, including what decision, by whom, made how, when, where, and why. Practices scoring well on this macrocognitive function had defined protocols for care management decisions, and specified where discussion with others was needed. For example, these practices had protocols in place for identifying eligible patients in the registry, for providers to refer patients to care management, and for scheduling appointments with care managers:

*“Interviewer: Tell me a little bit more about this stamp area; [and how] the stamp is supposed to remind you that this [patient] is an appropriate person [for care management].*

*Provider: We’ve set up the criteria such that; I think it may even be just a simple body mass index criteria plus if they’re a diabetic. I like the stamp because I look at it and I show the patient ‘I go you know this stamp means that you’re in trouble.’*

*Interviewer: So are there times when you see the stamp and you don’t address care management with that patient?*

*Provider: Yeah. Often because there are other issues going on that are frankly a higher priority at that time, and because I know that in my systemic process arrangement that the flow is such that if I disregard that stamp they’re still going to get the opportunity to schedule that care manager follow up. When my MA staff are checking them out, if I haven’t crossed off care manager referral, they’ll put in the care manager referral.”* [Physician in practice B]

When decision making was ineffective, there seemed to be confusion about who was supposed to be deciding what, what needed to be decided, or how. Some providers did not understand the type of patient best served by the care manager and sent inappropriate patients to the care manager. Other providers simply made blanket decisions that they would not refer any patients to care management. This was not set out of bounds in the planning process. Hence, this is an example of something that pertains to both a practice’s decision making and planning functions.

As this planning/decision making example illustrates, the macrocognition functions overlap and interact extensively. Another example appeared in the overlap between decision making and management of uncertainty, associated with patient billing. Patient insurance coverage for care management varied by insurance, and a given patient’s coverage was often not known to the provider at the time of the decision to offer care management to the patient. This became a source of confusion, frustration, and in some cases social justice for care managers, providers, and staff. Practices that successfully managed this issue had a policy of communicating the uncertainty of this situation to patients so that patients could make their own decisions about participating. Practices without a standing policy struggled with the decision on a regular basis.

### Managing the unknown

This function describes how uncertainty, risk, incomplete information, and unexpected events or findings are dealt with. A team’s abilities in managing the unknown are inevitably tested in launching a new program. Although thoughtful planning by an experienced team can help avoid many problems with implementation, some things cannot be known at the outset or cannot even be anticipated.

Practices that managed uncertainty well had a specific mental model of change: It always involved mis-steps and corrections, and that those were expected features of change rather than evidence of failure. A strategy often used by these practices was a “try it and tweak it” approach. They would try something on a very small scale (such as with one provider for one day), where the costs of the inevitable problems was low, then evaluate and adjust before rolling it out to other providers or continuing it for the long term. This strategy also incorporates the functions of monitoring and detection and sensemaking.

All practices faced a common uncertainty in not knowing how care management was performing financially and in terms of patient outcomes overall. They could look at the individual patient data, but there was no mechanism to pull all the data to see how their patients in care management were doing in absolute terms, or relative to patients in their own or comparison practices not receiving care management. Different practices had different understandings of and beliefs about this uncertainty. Most practices had compelling stories of patients who were responding positively to the care managers. For some providers and staff, this was enough for them to believe care management was of some benefit. Other providers and staff really wanted “hard” data to confirm that their efforts were resulting in patient improvement.

### Monitoring and detecting

Monitoring and detection consists of tracking implementation progress, as well as being actively open to novel or emerging situations that present potential opportunities or problems. The ability to identify an opportunity or problem accurately and early can be important to successful care management implementation. Monitoring and detection are often enacted through “Plan-Do-Study-Act” (PDSA) cycles and are essential macrocognitive functions for quality improvement (QI) activities. Practices employed both formal monitoring and detection processes, such as systematic evaluation, and informal ones, such as serendipitous noticing of problems or opportunities.

Practices that were effective at monitoring and detecting had well-developed, systematic means to identify patterns that could turn into problems. For example, in one practice the practice manager was concerned about the medical assistants (MAs) completing specific tasks related to support care management efforts. She developed an MA “report card” that MAs would complete to monitor their performance in completing these tasks so that non-completion of tasks would be identified.

Practices with good monitoring and detection function also made explicit provisions to support the informal means. One key feature was habitual conversations between care managers and staff, particularly regarding how the care management process was going and if things were “falling through the cracks.” Such conversations included ensuring that tests ordered were not left unordered, and issues identified by the care manager (such as a follow-up visit with the provider to modify dose of medication) were handled. A lot of what fell through the cracks occurred where there were information hand-offs between staff members. Some common examples included getting completed tests documented in the record, recording medication refills, arranging patient visits needed for regular maintenance, or referrals to classes or specialists. Practices with good monitoring and detection function caught these problems through habitual conversations and identified systemic problems by empowering staff with effective feedback mechanisms:

*“The way it works here, and it’s worked well I believe is there is a meeting agenda located on the door of the provider’s office where you can write those things on in terms of topics that need to be discussed during the meeting.”* [Care manager in practice D]

Those with poorer function had either structural (physical location of personnel) or habitual barriers impeding these conversations or did not actively seek or share feedback:

*“Interviewer: Have you received any feedback on how it’s going?*

*Reception Supervisor: Not really yet, no. No complaints, so I think usually no news is good news.”* [Reception supervisor from practice E]

### Sensemaking and learning

Sensemaking is a deliberate and systematic attempt to find coherent, conceptual *situational* understanding when faced with novel or ambiguous circumstances. In contrast, learning involves formal and informal mechanisms to acquire new knowledge or skill. Both involve deliberate efforts to create or modify a team’s mental models—its understanding of how processes do or should work, how the environment affects it, what actions produce what consequences, and through what mechanisms.

These two related functions drew the most from previous training on quality improvement processes. All the practices in the study had previously received training and had some previous experience with quality improvement using the Lean method (www.lean.org) in a learning collaborative facilitated by the Lean Enterprise Institute. Practices that did well at sensemaking applied the skills they gained from their Lean coaching to the implementation of care management; those that did poorly did not transfer the skills learned in the previous implementation to this one.

Another key difference between practices was the presence or absence of structures to support systematic sensemaking and learning. This included tools for monitoring and detection that deliberately triggered sensemaking and learning processes, and processes to review data and make collective informed decisions about what the data meant and how to modify workflows in response to it. Practices that did not have such sensemaking and learning processes and structures seemed to flounder when things were not working, and were not sure why. Even in cases when they did have a sense of why things were not working, they were ill-equipped to discover what to do to improve it. As with planning, turnover contributed to this problem by disrupting shared sensemaking.

Competing priorities also consumed the attention and resources of practice members, making it difficult for them to devote the time and energy needed to consider what was not working and how to fix it. A key cause was the implementation of a new EMR upgrade about 8 months into the care management implementation. Practices that had not already successfully worked out their care management processes from that change before the project started were derailed by their staff’s attention and resources being consumed with EMR issues.

Many of these functions were in place because the care manager or provider champion had a mental model of how things ought to be. For example, one of the most effective care managers we interviewed drew on her network outside the practice to learn what she needed to learn about care management:

*“The other office [where I shadowed] had a workflow. I took their criteria for initiating patients and planned care and adapted it to our office, and so then I kind of worked with what we had and kind of developed a workflow.”* [Care manager in practice B]

Similarly, one provider champion of care management had established a culture of change where everyone’s input was valued:

*“Dr. A has this term, leadership from the middle. You know, where he thinks that the people doing the work are the people who have the best ideas about how to get stuff done. Honestly, a lot of the times that we’ve develop protocols for various processes, not just this kind of stuff, but from office processes, back office workflows with the EMR and stuff, we do distribute it out and share it out to the rest [of the physician organization].”* [Physician in practice B]

## Discussion

In answer to our first research question, we found that the macrocognition framework was helpful in illuminating beliefs, processes, structures, and dynamics that were important in successfully implementing care management that is regularly used by practice members and their patients. The framework guided us to probe team functioning in areas of demonstrated importance. Its extensive track record in a range of industries and applications, and the fact that its core constructs have proven stable across investigations in that broad range [29–31], provided reasonable confidence that we were overlooking no major areas. It broke down “teamwork” into manageable and coherent constructs that were feasible to analyze in detail. It avoided the generation of analytic constructs idiosyncratic to this project or even limited to primary care, providing instead constructs that could be understood in the context of a broad multi-disciplinary cognitive and organizational science literature. The CTA methodology elicited rich detail about exactly how, and how well, macrocognitive skills were employed. It is not possible to gauge with certainty whether the CTA method allowed us to obtain data that would have not come to light otherwise, but the CTA family of techniques was developed with the express purpose of effectively uncovering tacit and dispersed knowledge that would otherwise be missed. Our participants noted enough “aha” moments in their interviews to suggest that that occurred in this project as well.

For our second research question, we found that differences in macrocognitive functions were associated with, and helped understand, observed differences in care management implementation success in the studied practices. For example, in the area of sensemaking and learning, use of CTA differentiated practices that could describe how they were able to identify a problem, seek to understand why the problem was happening, and how to go about fixing it. They had not only the cognitive understanding of this concept, but also the processes and structures to make it happen. Ineffective practices seemed to flounder when things were not working, were not sure why, and were ill-equipped to discover what to do to improve. Overall, in particular, differences in coordination, decision making, and sensemaking and learning differentiated practices that moved forward with implementation versus those that stalled.

These results add to the literature in two important areas. First, there are many papers intended to describe ways to implement care management [9, 13], but little in the literature about what explains effective care management implementation in practice. Daaleman et al. [12], in a study of clinical endpoints and surveys of clinician and practice staff member perceptions, note that “Physicians and care staff uniformly noted that outreach and personal communication by the care manager were key elements in effectively implementing the position into the FMC workflow.” Taliani et al. [9] studied practice-based care management implementation in 25 practices in southeastern Pennsylvania working toward improved diabetes care under PCMH. They used a positive deviance method to identify high and low performing practices, interviewed practice staff, and used a grounded theory methodology to analyze their data. Consistent with our results, they found that “upper-tercile care managers performed patient-centered duties, fully leveraged the potential of the EMR for communication, patient tracking, and information sharing; and had open and frequent communication with physicians and office staff. In contrast, lower-tercile care managers performed administrative duties, were unable to harness the communication and tracking potential of the EMR, and had less frequent intra-office communication” [9]. However, neither they nor others have set these findings into a larger theoretical framework that generalizes beyond primary care or medicine as does the macrocognition framework.

A second contribution to the literature is the use of the macrocognition framework in primary care. The methods associated with this framework have been utilized in many other fields to understand and improve processes, as noted in the Introduction, but are just emerging in primary care. Since primary care is both highly relational and involves complex cognitive work, the macrocognition framework and cognitive task analysis (CTA) tools offer promise for application to other new implementation processes in care settings.

A logical next step in care management implementation would be to design practice-specific interventions based on CTA findings to improve practices’ macrocognitive functions, and determine whether that improved their ability to implement care management. These could be conducted in conjunction with existing quality improvement processes.

One limitation of this study is its small sample of practices implementing care management. We only had the opportunity to study five practices in Michigan, although we did study those practices in depth over nearly a year. These practices represented two different primary care disciplines, and varied in size and rurality, which gives the findings some external validity. Our focus was to generate understanding about the question under study; in this case assessing the macrocognitive functions of teams and organizations that support effective implementation of care management.

Another limitation is this study’s focus on one theoretical perspective. We recognize that other theoretical perspectives may identify factors important in understanding successful implementation. Our intent in this paper is to examine the value that the macrocognition framework can provide, in understanding the processes teams can use to get implementation going (like planning) and getting unstuck (like sensemaking and learning). Understanding specific macrocognitive processes is likely to be helpful for teams attempting to implement care management, as it may shape their thinking about what they can do to move implementation forward. A detailed consideration of or comparison across other theories is beyond our scope here, but those familiar with the Consolidated Framework for Implementation Research [32, 33] will recognize that the macrocognition framework addresses most of the constructs and subconstructs of the “inner setting” and “implementation process” domains, and some constructs of the “individual characteristics” domain. CTA provides tools that can generate detailed understanding of the inner setting constructs and both understanding of and guidance for the implementation process.

From a practical perspective, our results in primary care combined with CTA’s track record in other knowledge work disciplines suggests that practice leaders may be able to use CTA to gain insight into where their teams may need support or improved skills to successfully implement care management. However, there is a corresponding limitation; CTA is a skill that requires substantial training. Trained personnel are in limited supply, as are those who can provide training for an organization that wishes to develop this capacity.

## Conclusions

Two conclusions can be drawn from this work. First, macrocognition is a useful framework for understanding some of the less visible functions that are important in care management implementation. Utilizing this framework offers an opportunity to get in deeper and understand specific elements of teamwork that support a new intervention in practice. Second, practices’ macrocognitive functions are closely associated with their care management implementation success. That suggests that practices seeking to implement care management may benefit from assessing their macrocognitive functions when instituting a new intervention such as care management. Practices that invest in planning how to implement are deliberate in making provisions for decision making and for monitoring and detection, that structure their coordination, and encourage and support sensemaking and learning, appear to be more successful with working through the processes necessary to make a new intervention work effectively and then “stick.” When these functions are attended to, our findings suggest that care management is more likely to become an integrated new intervention in practice. Difficulties in any one area should alert practice leaders to potential problems that may require additional actions to resolve them.
